# Performance variations among clinically available deformable image registration tools in adaptive radiotherapy — how should we evaluate and interpret the result?

**DOI:** 10.1120/jacmp.v17i2.5778

**Published:** 2016-03-08

**Authors:** Ke Nie, Jean Pouliot, Eric Smith, Cynthia Chuang

**Affiliations:** ^1^ Department of Radiation Oncology Rutgers ‐ Cancer Institute of New Jersey Rutgers‐Robert Wood Johnson Medical School New Brunswick NJ USA; ^2^ Department of Radiation Oncology University of California San Francisco CA USA

**Keywords:** deformable image registration, contour transformation, dose warping, adaptive radiation therapy

## Abstract

The purpose of this study is to evaluate the performance variations in commercial deformable image registration (DIR) tools for adaptive radiation therapy and further to interpret the differences using clinically available terms. Three clinical examples (prostate, head and neck (HN), and cranial spinal irradiation (CSI) with L‐spine boost) were evaluated in this study. Firstly, computerized deformed CT images were generated using simulation QA software with virtual deformations of bladder filling (prostate), neck flexion/bite‐block repositioning/tumor shrinkage (HN), and vertebral body rotation (CSI). The corresponding transformation matrices served as a “reference” for the following comparisons. Three commercialized DIR algorithms: the free‐form deformation from MIMVista 5.5 and the RegRefine from MIMMaestro 6.0, the multipass B‐spline from VelocityAI v3.0.1, and the adaptive demons from OnQ rts 2.1.15, were applied between the initial images and the deformed CT sets. The generated adaptive contours and dose distributions were compared with the “reference” and among each other. The performance in transferring contours was comparable among all three tools with an average Dice similarity coefficient of 0.81 for all the organs. However, the dose warping accuracy appeared to rely on the evaluation end points and methodologies. Point‐dose differences could show a difference of up to 23.3 Gy inside the PTVs and to overestimate up to 13.2 Gy for OARs, which was substantial for a 72 Gy prescription dose. Dosevolume histogram‐based evaluation might not be sensitive enough to illustrate all the detailed variations, while isodose assessment on a slice‐by‐slice basis could be tedious. We further explored the possibility of using 3D gamma index analysis for warping dose variation assessment, and observed differences in dose warping using different DIR tools. Overall, our results demonstrated that evaluation based only on the performance of contour transformation could not guarantee the accuracy in dose warping, while dose‐transferring validation strongly relied on the evaluation endpoint. As dose‐transferring errors could cause misinterpretations when attempting to accumulate dose for adaptive radiation therapy and more DIR tools are available for clinical use, a standard and clinically meaningful quality assurance criterion should be established for DIR QA in the near future.

PACS number(s): 87.57

## I. INTRODUCTION

It is acknowledged that patient anatomy may change during the course of radiation therapy due to factors such as weight loss, tumor and normal tissue growth or shrinkage, and intratreatment position variations.^1^, ^2^, ^3^, ^4^ For example, patients with head and neck cancer tend to lose weight during the treatment course; a consecutive shrinkage of the gross tumor volume of up to 3.9% per treatment day has been observed.^(3)^ Anatomical changes between the primary treatment and the retreatment are another situation that might be even more obvious. Cranial spine irradiation patients might be treated in the prone position and receive an additional spine boost with intensity‐modulated radiotherapy (IMRT) or stereotactic body radiotherapy (SBRT) in a supine position on TomoTherapy or CyberKnife (Accuray Inc., Sunnyvale, CA). The vertebral body shape could change and so can the organs around it. Although advanced treatment techniques such as IMRT and SBRT can establish conformal dose distribution and a sharp dose gradient, accurate information on the spatial distribution of the previously applied dose is essential for an effective and safe treatment of the recurrent tumor. Deformable image registration (DIR) provides the possibilities for linking the anatomy at one time to that at another time, while maintaining the desirable one‐to‐one geographic mapping. For this reason, there is increasing interest in bringing DIR within the context of radiotherapy.

In general, there are two main avenues using DIR in adaptive radiation therapy: a) to generate contours for efficient recontouring purpose or as a quantitative indicator of the need to perform replan, and b) to generate dosimetric plans for an adaptive plan summation to evaluate over‐/ underdose estimation of the accumulated dose. Previous studies to estimate the inherent accuracy of DIR have typically been performed only by comparing locations of anatomic landmarks or contours identified by physicians.^5^, ^6^, ^7^, ^8^ A recent work published by Hoffmann et al.^(5)^ evaluated the feasibility of a single commercial tool (Velocity AI, Velocity Medical Solutions, Atlanta, GA) in head and neck, thoracic and abdominal cases. A total of 30 to 50 landmarks were placed by physicians for a whole 3D volume for each case. The registration error was reported using 3D Euclidean distances between the corresponding landmarks in each CT pair. This regional point‐based analysis could be reasonable if evaluating solely the capability in transferring the contours. However, in addition to anatomy deformation, the dosimetric effect thereof is another important aspect. It is thus necessary to perform voxel‐by‐voxel analysis, as any unreasonable deformation might create erratic and artificial cold/hot spot or under/over dosage of the target and critical structures. There are several groups working on validating DIRs based on voxelized analysis derived from deformation vector fields (DVFs). Saleh et al.^(7)^ proposed a statistical sampling technique (distance discordance metric) to estimate the spatial geometric uncertainty. Similarly, Li et al.^(9)^ proposed to use a mechanics‐based metric, using unbalanced energy (UE) to evaluate DIR errors. Meanwhile, Varadhan et al.^(10)^ proposed several metrics, such as inverse consistency error (ICE) and mean squared error (MSE), to test deformation accuracy. Consent has been achieved that evaluation should be performed on a voxel‐by‐voxel basis. However, the clinical interpretation of all these proposed mathematical terms is missing and how these matrices would aid in making clinical decision needs to be addressed.

Very recently, Veiga et al.^(11)^ evaluated the performance of several in‐house implemented DIR algorithms for dose warping on five head and neck patients. Their findings concluded that in spite of all algorithms resulting in comparable geometric matching, the choice of DIR implementation leads to uncertainties in dose warping for head and neck cancer. As several commercialized DIR tools are already available for clinical use,^5^, ^12^, ^13^, ^14^ there is an emerging and urgent need to investigate the reliability of these clinical tools in multiple applications in daily clinical routine. Previously, we have compared deformed vector fields of two DIR algorithms on both physical phantoms and virtual patient images.^(13)^ Here, we extend our work to evaluate three commercially available DIR solutions for both contour propagation and dose matching/ warping using clinical examples, and further explore the possibilities to interpret the results using clinical language.

## II. MATERIALS AND METHODS

### A. Patient data

Three clinical plans were included:

Case #1 — a prostate cancer patient received 180 cGy for 25 factions (fx) for the first course of IMRT treatment. Patient had noticeable bladder filling variations during the treatment.
Case #2 — a head and neck (H&N) patient who lost nearly 40 lbs (22% of original weight at admission) during the course of the treatment, requiring the acquisition of a second set of CT images for replanning. The initial IMRT plan included three PTVs with prescription dose of 6996, 5940, and 4950 cGy over 33 fractions (PTV70, PTV60, and PTV50, respectively).
Case #3 — a patient with a cranial spinal irradiation (CSI) treatment in prone position who received 180 cGy / fx for 20 fractions, plus a L‐spine and posterior‐fossa boost in supine position.

For all treatment plans, standard OAR limits were used and dose was minimized to the OARs without reducing coverage of the targets.

### B. Reference for comparison

**Figure 1:**
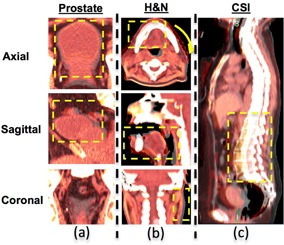
The virtual deformed images for the three clinical cases. Each of the images represents only one slice of axial, sagittal, and coronal view of a 3D CT data set. For CSI case, only the sagittal view is shown. The original and simulated deformation images are fused together to show the difference in deformation (highlighted in the yellow box): (a) prostate case with bladder filling, (b) head and neck case with significant weight loss, deformation includes head twisted by a small angle, relative smaller oral cavity closure even with a bite‐block, and noticeable narrower neck region; (c) cranial spinal irradiation case at prone position first then with lower spine IMRT boost at supine position with less curved spine.

For comparison purposes, computerized synthetic deformed CT images were created from the planning CT images for each case. The synthetic deformation was driven by a fiducial‐based algorithm provided by a commercial QA software tool (ImSimQA, Oncology Systems Limited, Shrewsbury, UK). The details of how to generate synthetic deformations and how to validate those movements were introduced in Nie et al.^(12)^ To recap briefly, ahead of utilization of this QA tool, this computerized deformation algorithm was validated using an in‐house‐built physical pelvic phantom.^(15)^ For the real patient cases used in the study, planning CT sets for the same patient at different treatment time points were firstly aligned with each other. For each image set, approximately 30 anatomically meaningful landmarks were carefully chosen by a physician. For the prostate case, the patient with bladder filling could result in an average bladder inflation of 6.4±3.7 (standard deviation, (SD)), ranging from 0.0 to 19.5 mm. The significant weight loss for the head and neck case could result in anatomical point displacements of up to 5.1±3.0 (0,15.5) mm. The cranial spinal patient was first treated in a prone position and three months later in a supine position with a whole brain and lower spine boost. On average, the anatomical landmarks at the skull, temporal lobe, and spine had changes up to 34.7 mm (with an average of 8.8±4.2 mm) for retreatment. Detailed information can be checked in our previous work.^(12)^ Over all, the corresponding displacements of all the paired points were recorded to provide the possible extent of movement between the two treatments. To generate synthetic deformed images, 20 to 50 control points for each case were selected by an experienced user on the initial planning CT images. The user also identified the moving direction and displacement for all the points. After that, the synthetic moved images were generated using the software tool with either stiff deformation option (for bony structure) or not (for soft tissue). The initial planning CT images and the synthetic deformed images are shown in Fig. 1. Overall, the generated deformations included: 1) bladder filling for the prostate case, 2) head rotation along the inferior—superior axis, neck flexion change, weight loss in the neck region, and smaller oral cavity for the HN case, and 3) more straightened spine for the supine position and soft‐tissue movement as in lung and bowel for the CSI case. In addition, Gaussian‐type noises were added to the deformed CT images to make the initial and deformed CTs be more representative of two distinct images. The level and strength of the noises matched with results obtained from experiments using size‐comparable water phantoms.^(12)^ The resultant deformed image sets, contours, dose plans, and the corresponding deformed vector fields were exported and served as the “reference”.

### C. Deformable image registration procedure

Three commercial software packages were evaluated, which included (a) the free‐form deformation — MIM (both MIMVista v 5.5 and Reg‐Refine MIM Maestro v 6.0) (MIM Software Inc., Cleveland, OH), (b) the B‐spline deformation — Velocity AI (v 2.6.2) (Varian Medical Systems/Velocity Medical, Atlanta, GA), and (c) the adaptive Demons —OnQ rts (v 2.1.15) (Oncology Systems).

The free‐form deformation from MIM, v 5.5, is a fully automatic intensity‐based deformation with a multiresolution approach.^(16)^ The most updated version, v 6.0, introduces a new feature, RegRefine, to allow certain level of freedom for users to repeatedly lock and correct the registration. The number of lock points can be varied in addition to varying their locations and the size of the window used for local box‐based alignment. For practical purposes, three to five constraint points were selected for each case by an experienced operator to assure deformation accuracy using visual assessment. VelocityAI uses a modified B‐spline based calculation combined with the Mattes formulation of the mutual information metric.^(17)^ For the multipass option, the grid resolution spacing starts at the coarsest and goes down to the finest in multiple steps, which helps to speed up the convergence and avoid local minima. As recommended by the vendor, the registration type “rigid and deformable multipass” was chosen. The OnQ rts is the product from the same company as ImSimQA, yet it uses a stand‐alone deformable algorithm as adaptive Demons. The registration process is a fully automatic process, which starts with rigid alignment and then follows with deformable registration. For all the commercial software tools, information on the details of the parameter used in the software is not provided by the vendor nor can the user change parameter settings for deformation, thus the default registration settings were used throughout the study.

In a clinical dose escalation situation, the total plan is typically generated as the summation of the initial treatment plan delivered to the initial CT and boost plan delivered to the second CT. If the two image sets have noticeable variations, DIR may be requested to prepare for the adaptive plan sum. As such, in this study, all available DIR tools were used to deform initial planning CT images (moving images) to match the synthetic deformed CT images (target images) created by ImSimQA. A region of interest (ROI) was defined in the primary volume to exclude any external positioning and fixation devices in order to avoid their influence on the registration process. Attention was given to ensure all defined relevant anatomical structures were included. The resulting deformed contours, dosimetric deformed plans, and all calculated vector fields were exported and compared against each other with respect to the “reference” using an in‐house MATLAB program (MathWorks, Natick, MA).

### D. Evaluation procedure

Both the contour propagation and dose transformation capabilities were evaluated.

1. The contour transferring accuracy was assessed using Dice similarity coefficient index over the entire 3D contoured volume. The index is defined as twice the intersection divided by the union sum of two volumes. A perfect match gives a Dice index of one and an absolute mismatch results in a zero. In this study, for all the PTVs and OAR structures, Dice coefficient indices were calculated between the “reference” contour and that transferred, via different DIR tools, from the moving images to the target images.
2. The adaptive plan was made by applying the deformed vector fields to the original dose for the whole course. The dosimetric impact was evaluated following the steps that physicians typically use to evaluate plans. Firstly, the dose‐volume histograms (DVHs) for all the structures generated using different DIR tools were compared against each other. Secondly, two‐dimensional (2D) dose distribution was qualitatively assessed by comparing with the reference plan and against each other on a slice‐by‐slice basis. Thirdly, point‐dose differences inside PTVs and all OARs were compared to check whether unreasonable point doses were created. All point doses were calculated requiring a minimum volume of 0.5 cc. In addition to all these evaluations as used in clinical assessment, quantitative analysis using three‐dimensional (3D) gamma index comparison was investigated. The 3D gamma index was calculated following the method presented by Wendling et al.,^(18)^ and the passing rates were calculated with various testing criteria.

## III. RESULTS

### A. Contour warping evaluation

Because very minimal differences were observed from different versions of MIM software in the selected three cases, only results from the fully automatic method (MIM v 5.5) were presented here and compared to others. Table 1 summarizes the Dice coefficients of all structures. All three algorithms showed reasonably comparable results in warping contours but performances varied in selected examples. For instance, Dice coefficients for all PTVs / OARs transferred by Tool#1 and Tool#3 ranged from 0.76 to 0.98, with an average of 0.87 for prostate and HN case. However, Tool #3 showed issues with prone‐supine deformation and slice thickness beyond 0.5 cm as in the CSI case, with the lowest Dice index of 0.55.

**Table 1:** Dice coefficients of all adaptively transferred contours vs. references, showing with mean ± SD (minimum, maximum)

	*Prostate*		*HN*		*CSI*	
	*Target*	*OARs*	*Target*	*OARs*	*Target*	*OARs*
Tool#1	0.94±0.02	0.97±0.01	0.94±0.02	0.90±0.05	0.89±0.03	0.92±0.07
	(0.92,0.96)	(0.96,0.98)	(0.91,0.95)	(0.78,0.96)	(0.88,0.91)	(0.75,0.97)
Tool#2	0.79±0.18	0.87±0.05	0.79±0.13	0.73±0.14	0.83±0.07	0.89±0.05
	(0.78,0.92)	(0.83,0.92)	(0.70,0.88)	(0.42,0.89)	(0.78,0.87)	(0.77,0.98)
Tool#3	0.85±0.10	0.89±0.05	0.92±0.03	0.88±0.06	0.58±0.04	0.70±0.18
	(0.79,0.96)	(0.84,0.94)	(0.89,0.94)	(0.76,0.92)	(0.56,0.61)	(0.55,0.92)

**Figure 2:**
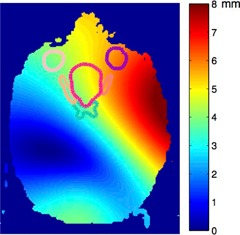
The composite deformation vector field (DVF) field from synthetic H&N phantom, with outlined contours of PTV and organs at risk (OARs) as eye (left/right), optical nerve (left/right), and chiasm. Color represents the magnitude of the deformations, with red meaning more deformation and blue, less deformation.

Tool#2 showed an overall good agreement with the “reference” for prostate and CSI cases. But noticeable differences were observed in the HN case. Figure 2 gives the composite deformation vector field (DVF) of one axial slice showing the left anterior side with larger displacement compared to the right posterior region. The low matching numbers of contour warping were more inclined to show on the side with larger displacement (e.g., cochlea left vs. right (0.42 vs. 0.61), and temporomandibular joints (TMJ) left vs. right (0.62 vs. 0.85) and eye left vs. right (0.60 vs. 0.75)).

### B. Dose transferring evaluation

#### B.1 Volume‐based analysis — DVH comparison

**Figure 3:**
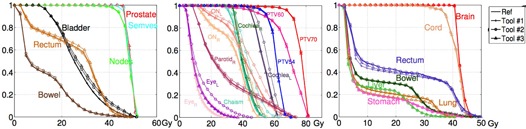
DVH analysis for three cases: prostate, HN, and CSI, respectively. The “reference” is displayed in bold solid line. The result for Tool#1 is shown in line with +; Tool#2 shown in line with ∘, while Tool#3 result is shown in line with Δ.

The coverage of the PTV by 100% of prescribed dose line (prostate/seminal vesicle/nodes for prostate case, PTV 70/PTV 60/PTV 54 for HN case, and boosted L‐spine/brain for CSI) showed no difference across the three applied DIR algorithms, as shown in Fig. 3. Results also showed very close DVH lines for all the tested OARs. Exceptions were only noticed for Tool#2 results on the HN case. Even though Tool#3 showed low matching numbers of Dice indices for the CSI case, the DVHs did not reveal much variation compared to others. Barely visible differences could be seen for deformation on images with or without (w/o) adding Gaussian‐type noises.

#### B.2 Slice‐based analysis — Two‐dimensional (2D) dose plane comparison

**Figure 4:**
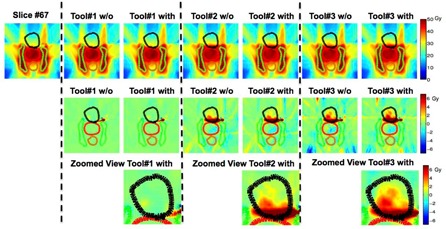
One axial slice view showing the prostate case among all three algorithms without (w/o) or with adding Gaussian‐type noise. All contour colors match with corresponding DVHs in Fig. 3. The absolute dose distribution is shown on the top row, while dose difference in Gy compared to the “reference” is shown on second row with a zoomed view of the prostate (PTV) region on the bottom.

**Figure 5:**
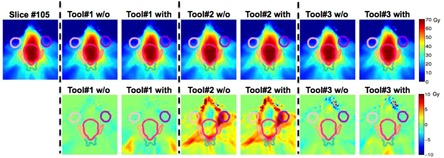
One axial slice view showing the warped absolute dose HN case among all three algorithms.

**Figure 6:**
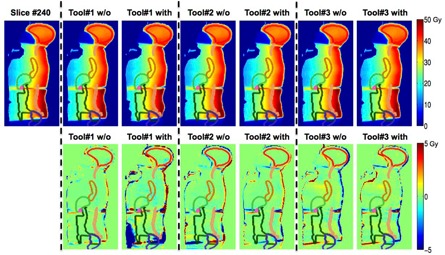
One sagittal slice view showing the warped absolute dose and dose difference in Gy for cranial spinal irradiation (CSI case) compared to the “reference” for all three algorithms with all contour colors matching with corresponding DVHs in Fig. 3.

We further scrolled through all two‐dimensional dose planes slice‐by‐slice. One representative slice for each case is shown in Figs. 4 to 6 with all the contours colors matched with Fig. 3. The absolute dose distributions are shown on top row, with or without (w/o) adding Gaussian‐type noises and the respective dose differences to the “reference” are displayed on the second row. In general, Tool#1 gives the closest adaptive dose plan to the reference for the three selected cases. Although the DVH lines show small differences between Tool#3/Tool#2 and Tool#1 for the bladder (contour in black) in the prostate case, the slice‐by‐slice regional analysis for Tool#2/Tool#3 show up to 6 Gy differences compared to Tool#1, which can be substantial if considering the 45 Gy prescription dose.

For the HN case shown in Fig. 5, Tool#1 and Tool#3 present comparable performance. However, Tool#2 shows up to 10 Gy differences in multiple OAR regions, as high as 14% of the prescribed 70 Gy. The differences were laid mostly on large displacement regions, such as on the left side and the oral cavity region. The results for the CSI case are presented in Fig. 6. Tool#1 and Tool#2 give similar deformations, but not with Tool#3. Regarding the noise effect, only Tool#1 exhibited visible variations between the results deformed to images with and without Gaussian‐type noise. Those differences were more likely to show in the low‐dose and low‐gradient areas such as in the lower abdominal regions for the CSI case. However, these might not have clinical impact if regions were far away from clinically relevant organs exposed to high doses.

#### B.3 Point‐dose analysis

**Figure 7:**
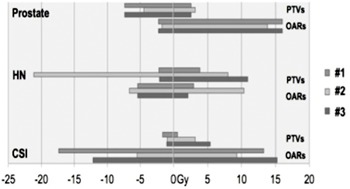
A range of point‐dose differences compared between the transformed dose via different DIR tools and the reference dose.

Figure 7 shows the point‐dose differences relative to the “reference” of all three algorithms. It can be seen that Tool #2 has the largest point‐dose differences compared to the “reference,” as expected. Interestingly, even Tool#1, the algorithm showing the highest Dice coefficient, exhibited point‐dose differences up to 7.7 Gy inside the PTVs for prostate case. It also overestimated up to 13.5 Gy and underestimated by up to 17.7 Gy in OARs for CSI cases.

#### B.4 Quantitative three‐dimensional (3D) analysis — 3D gamma index comparison

**Figure 8:**
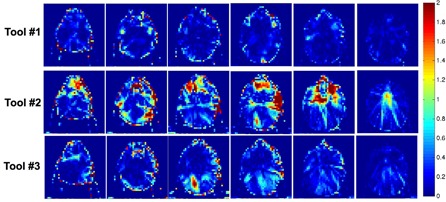
Selected series of slices showing the gamma index map on the axial view for the head and neck case using 3 mm/3% criteria.

The equivalency between the tested algorithms and the “reference” was further verified using a quantitative 3D gamma index test. Table 2 summarizes the results using 3 mm/3% (distance‐to‐agreement / dose difference) or 5 mm/5% criteria. Although no plans exhibited significant differences in DVH comparison, not all of them had over 90% voxels passing the 3%/3 mm criteria. For the HN case, Tool#2 had only 72.7% of the voxels meet the criteria. For the CSI case, Tool#3 showed an 86.1% passing rate. An example of a gamma index map of an axial plane for the HN case is given in Fig. 8. If using 5 mm/5% as the criteria, all algorithms passed with less than 10% of the voxels exceeding the criteria.

**Table 2:** Three‐dimensional (3D) gamma analysis for different DIR algorithms

	*3 mm/3%*			*5 mm/5%*		
	*Prostate*	*HN*	*CSI*	*Prostate*	*HN*	*CSI*
Tool#1	97.7%	96.1%	93.4%	99.4%	98.9%	95.7%
Tool#2	93.1%	77.2%	94.1%	94.8%	90.7%	94.5%
Tool#3	93.4%	94.7%	82.4%	96.3%	97.4%	93.1%

## IV. DISCUSSION

In this study, we evaluated the performance variations of three commercially available DIR tools for three different clinical cases. These three algorithms produced comparable results transforming contours based on Dice coefficient analysis, and have similar results in dose warping when assessed by volume‐averaged DVH analysis. However, dose‐warping performance exhibited noticeable differences if the dose map was reviewed slice‐by‐slice or analyzed with 3D gamma‐index comparison. In addition, there is no consistency in performance for the DIR algorithms since the performance varied with clinical scenarios. Some tools might excel in certain situations but not in another. Therefore, our study showed that validation of DIR tools should be performed patient‐specifically and a standard acceptance term and criterion should be established for the evaluation.

Previous literature has highlighted the need for validation of the DIR tools, including large‐scale, multi‐institutional studies.^19^, ^20^ Yet there are still remaining questions to be answered, specifically on how to evaluate and how to interpret the results.

DIR is generally used for time‐efficient recontouring or adaptive dose‐accumulation to optimize therapeutic efficiency. As such, for the first question of “how to evaluate,” assessment of both capacities, contour propagating and dose tracking, should be included. The current techniques for DIR evaluation generally fell into three categories: contour comparison,^21^, ^22^, ^23^, ^24^, ^25^ landmark tracking,^5^, ^23^ and voxel‐based analysis.^7^, ^8^, ^9^, ^10^, ^12^, ^13^ Both the contour‐ and landmark‐based analyses can yield a skewed view of the overall registration accuracy, as they only test spatial accuracy in limited regions. We believe voxel‐based analysis should be utilized as it provides information that is more comprehensive. Yet, “how to interpret” this massive 3D information is another issue. Publications in the literature proposed several scientific terms, such as Jacobian matrices,^(26)^ inverse consistence error,^(10)^ and unbalanced energy,^(9)^ to test deformation accuracy. Those terms may illustrate the physical properties of the deformation, but they still yield certain levels of difficulties for clinical understanding. Here the possibilities to use clinically oriented terms is explored, following the steps physicians usually take to evaluate the plans, as contour propagation, then dose coverage of PTVs, dose sparing for OARs, slice‐by‐slice review, and DVH comparisons. As illustrated in this study, different observation points/methodologies might give different results.

Firstly, point‐based analysis might present oversensitive skewed information as even the most accurate algorithm in transferring contours or dose would show nontrivial point‐dose differences inside of all structures. On the contrary, volume‐based analysis such as DVH comparison might not be a sensitive enough measurement to reveal differences. For example, visible dose variations could be located inside the bladder region for the prostate case and for lower abdominal areas for the CSI case, but DVH differences were very subtle. DVH analysis might only capture the difference when it was distinctive enough, as in the HN case. Furthermore, 2D slice‐by‐slice review could help in revealing the extent of difference in details. Still, it was a time‐consuming process and provided only a qualitative assessment.

So for our analysis, we explored the possibility using the well‐established gamma index concept for quality assurance adapted for the DIR evaluation. The results showed if using 3 mm/3% as the acceptance level, all three algorithms presented reasonable levels of equivalency. Only Tool#2 had over 10% of the voxels not meeting this criterion for the HN case and Tool#3 failed for CSI case. Further studies on whether we could adopt this concept for DIR QA, how to use it either in traditional planar 2D or 3D analysis, and how to setup a standard acceptance level such as 3 mm/3% or use another criterion warrants more investigation.

Additionally, the DIR performance varies with different situations. Tool#3 presented similar performance to the other two tools in the prostate and HN case, but it revealed some issues in transferring prone/supine position as in CSI case. The vendor has been informed of this and a newer version is being released to correct this issue. Further evaluation is needed for this particular situation. Tool#2 exhibited equivalent results compared to others, but not for the tested HN case. The low matching numbers were more likely to be shown in the regions with large displacement, such as along the left side and oral cavity. There are usually two items regulating the deformation: image similarity and deformation smoothness. The first term controls the similarity based on pure mathematic considerations of either or combined information from intensity, gradient, and many other high‐ordered functions. On the other hand, the deformation smoothness controls the realism of the deformation and avoids extreme and unreasonable physical deformations. For the tested HN case, Tool #2 might emphasize the deformation smoothness more over the similarity, thus the variations were shown on the large displacement side. A research version with the capability of adjusting the weightings of these two regulation terms for various scenarios is under investigation. In addition, although we have tried to generate synthetic deformations covering most of the clinical scenarios for each site, such as bladder filling for the prostate case; head twist neck flexion, and other changes due to weight loss for the head and neck case; and less curved spine due to the change from prone to supine position, we still cannot cover every aspect for each location. We only want to exhibit the performance variations of commercially available DIR tools using three clinical examples and further to discuss the importance of evaluation criterion in assessing those differences. Nevertheless, comparison or validation of those DIR tools should be performed for different scenarios, and in challenge cases, a patient‐specific test might also be needed.

It has to be pointed out that the purpose of this study is not to identify the most accurate algorithm, due to the absence of ground‐truth deformations. The ground‐truth deformations are typically derived from either physical image/dosimeter phantoms^15^, ^27^, ^28^, ^29^, ^30^ or synthetic computerized data.^5^, ^26^ Of note, it is unrealistic to design physical phantoms to simulate every clinical scenario. Synthetic virtual phantoms derived from actual patient image data may provide a complementary work. However, virtual deformation is still driven by computerized algorithms. Nonbiomechanical algorithms, which do not consider the elastic properties of tissues, may produce synthetic deformations in nonphysical ways. Biomechanical algorithms, potentially more accurate, are also likely to have errors due to uncertainties in Young's modulus and Poisson's ratio inputs. Overall, cross‐calibration of the virtual phantom with a physical phantom or patient‐similar tissue is needed before wide utilization for validation process; but this is beyond the scope of the current study. In this study, a commercial QA software tool was used to generate synthetic deformations that derived directly from patient‐planning CT images. This approach gave the possibility of evaluating various clinical scenarios for different sites. In our previous work, the QA software that generated virtual deformations was partially validated using a physical pelvic phantom.^(13)^ Although the physical phantom was created from real patient CT images with three heterogeneous soft tissue types as well as bone, this 2D phantom did not contain three‐dimensional information or materials to mimic air HU to simulate gas filling. It also should be noted that the pelvic phantom was designed only for bladder‐filling simulation. As it is not feasible to have various kinds of physical phantoms to represent every possible deformation, we did not extend our work to validate virtual deformations nor validate the accuracy of the DIR tools. We did not rely on the virtual deformations as ground‐truth to check the DIR accuracy, but only used them as a third‐party “reference” to explore the performance variations among each other.

It is understood that the clinical application of dose‐warping technique is a contentious topic as reflected by, for instance, a point‐counter point article and correspondences published by *Medical Physics*.^31^, ^32^, ^33^ Although the answer is not likely to be without complexity, a number of published studies have shown the applicability of dose‐warping technique. Yeo et al.^(29)^ demonstrated an experimental validation of the dose‐warping technique and showed it could be justified for those not involving significant density changes. The related discussion is beyond the scope of this paper. As DIR tools are being utilized to facilitate the calculation of cumulative doses over different states of deformation and the commercially available DIR tools have emerged rapidly in the field, we need to know how many differences those tools will present for various clinical situations, and at what level we can rely on them for clinical decision‐making.

## V. CONCLUSIONS

In summary, performance among three commercially available DIR tools in multiple clinical sites, using real patient images were compared. Their variations were evaluated in both capabilities, as contour transferring and dose tracking, and the results were interpreted using clinical language. The performance of these algorithms varied and is related to factors including tissue deformation magnitudes, dose gradient across the regions of interest, and also the evaluation standards. Nevertheless, as more DIR tools are available for clinical use, the performance could vary at certain degrees; a standard quality assurance criterion with clinical meaning should be established for DIR QA, similar to the gamma index concept, in the near future.

## ACKNOWLEDGMENT

The authors would like to thank Oncology Systems Limited, UK, for their support in providing ImSimQA for the duration of our research project.

## COPYRIGHT

This work is licensed under a Creative Commons Attribution 4.0 International License.
